# Trends in the Incidence of *In Situ* and Invasive Cervical Cancer by Age Group and Histological Type in Korea from 1993 to 2009

**DOI:** 10.1371/journal.pone.0072012

**Published:** 2013-08-16

**Authors:** Chang-Mo Oh, Kyu-Won Jung, Young-Joo Won, Aesun Shin, Hyun-Joo Kong, Jae Kwan Jun, Sang-yoon Park

**Affiliations:** 1 Cancer Registration and Statistics Branch, National Cancer Control Institute, National Cancer Center, Goyang, Korea; 2 Molecular Epidemiology Branch, Research Institute, National Cancer Center, Goyang, Korea; 3 Cancer Early Detection Branch, National Cancer Control Institute, National Cancer Center, Goyang, Korea; 4 Center for Gynecologic Cancer, National Cancer Center Hospital, National Cancer Center, Goyang, Korea; Vanderbilt University Medical Center, United States of America

## Abstract

**Objective:**

Our study aims to describe changes in carcinoma *in situ* (CIS) and invasive cervical carcinoma (ICC) in Korean women diagnosed between 1993 and 2009.

**Methods:**

All cases of CIS and invasive cervical carcinoma diagnosed from 1993 to 2009 in the Korean National Cancer Incidence database were analyzed. Age-standardized rates (ASRs) and annual percent changes (APCs) in incidence rates were compared according to age and histological type. Additionally, we used Korea National Health and Nutrition Examination Survey (KNHANES) to know the association between screening rate for cervical cancer and incidence rate of cervical cancer.

**Results:**

Between 1993 and 2009, 72,240 cases of ICC were reported in Korea. Total incidence rate of ICC was 14.7 per 100,000 females. ASRs of ICC declined 3.8% per year, from 19.3 per 100,000 in 1993 to 10.5 per 100,000 in 2009. Although the overall incidence rate of adenocarcinoma remained stable, invasive squamous cell carcinoma showed a decreasing trend (APC −4.2%). For women aged 60–79 years, ASRs for squamous cell carcinoma increased from 1993 to 2001, and decreased from 2001 to 2009 (APC: −4.6%). Total 62,300 cases of CIS were diagnosed from 1993 to 2009. Total incidence rate of CIS was 12.2 per 100,000 females. ASRs of CIS increased 5.7% per year, from 7.5 per 100,000 in 1993 to 19.0 per 100,000 in 2009. Adenocarcinoma *in situ* increased 13.2% per year. There was a strong positive correlation between screening rate for cervical cancer and incidence rate for CIS (*p*-value = 0.03) whereas screening rate showed a strong negative correlation with incidence rate for squamous ICC (*p*-value = 0.04).

**Conclusions:**

The increasing trend in CIS, coupled with a decreasing trend in ICC, suggests the important role of cervix cancer screening. The incidence of adenocarcinoma showed a plateau, but the incidence of adenocarcinoma *in situ* showed an increasing trend.

## Introduction

Burden of invasive cervical cancer ranked as the third most common cancer and burden of the mortality of cervical cancer ranked as the fourth in women worldwide [1], [2]. However, the incidence rate of cervical cancer showed a great variation between different regions, reflecting the human papilloma virus (HPV) prevalence [3] and screening rate of cervical cancer [4]. Although the burden of cervical cancer incidence has ranked as the second in Eastern Asian countries [2], the incidence rate of cervical cancer has decreased in Korea [5], China [6], and Taiwan [7].

Adaptation of screening programs have led to decreased incidence of overall invasive cervical cancers, especially squamous cell carcinoma in Europe [8] and the United States [9]. On the other hands, the recent increasing trends in cervical cancer incidence observed in Bulgaria and Romania were attributed to the insufficient or absence of cervical cancer screening programs [10]. However, some reports have indicated that the incidence of adenocarcinoma has increased not only among young women in the United States [11], Canada [12], and the Netherlands [13] but also among the entire population in the United States [9] and European countries [14], although cervical cancer screening programs were implemented.

Thus, cervical cancer screening was also suggested to have not sufficient effect on reduction of the incidence rate of adenocarcinoma [15], [16]. However, both squamous cell carcinoma and adenocarcinoma of the cervix showed a decreasing trend in all age groups in Taiwan after a nationwide cervical cancer screening program was introduced [7].

In this study, we investigated the trends in both carcinoma *in situ* (CIS) and invasive cervical cancer (ICC) by age group and histological type in Korea from 1993 through 2009 by using national registry data.

## Materials and Methods

### Data Sources

We analyzed the cervical cancer data from the Korea Central Cancer Registry (KCCR) and the gynecologic cancer registry. The ministry of Health and Welfare initially launched the KCCR as a nationwide, hospital-based cancer registry in 1980. The KCCR covered the whole population under the Population-Based Regional Cancer Registry program since 1999 [5]. The KCCR data from 1999 to 2002 have been published as Cancer Incidence in Five Continents, which reflects the completeness and validity of the incidence data [17]. The KCCR has constructed the Korea National Cancer Incidence database (KNCI DB) by merging the KCCR database and all 9 population-based regional cancer registry databases, the site-specific cancer registry databases, data from additional medical record review surveys, and the cancer mortality database.

For the gynecologic cancer registration, the Gynecologic Oncology Committee of the Korean Society of Obstetrics and Gynecology has operated a gynecologic cancer registry since 1991 [18]. These gynecologic cancer registration data since 1993 was checked and classified by the committee members who were all board-qualified clinical oncologists and pathologists [19]. Using these two databases, we can estimate the national cervical cancer incidence since 1993 [18].

In addition, to know the temporal trends of cervical cancer screening by age group, we additionally used Korea National Health and Nutrition Examination Survey (KNHANES) form 1998 to 2009 which had the complex random sampling survey design. KNHANES could be representative nationally for Korea. Data structure of KNHANES was described previously in detailed [20].

### Classification of Cervical Cancer

All cases among women with cervical cancer or CIS were selected from the KNCI DB. The information available comprises demographic characteristics of the patients, date of diagnosis, primary site of the tumor, and histologic type of the tumor according to the International Classification of Diseases for Oncology, 3rd ed. (ICD-O-3) [21]. For the histologic type, we classified three categories as follows: squamous cell carcinoma (ICD-O-3: 8010, 8052–8078, 8083–8084), adenocarcinoma (ICD-O-3: 8140–8147, 8255–8384, 8480–8772) cell types, and other and unspecified cell type [22]. We classified adenosquamous carcinoma, adenocarcinoma with squamous differentiation, mucoepidermoid and adenoid cystic carcinomas as adenocarcinomas.

### Definition of Screening Rate for Cervical Cancer

Screening rate for cervical cancer was defined as the proportion of participants who undergone in cervical cancer screening within 2 years [23].

### Statistical Analysis

Patients were divided into the following groups according to age: 20–29, 30–39, 40–49, 50–59, and 60–79. Age-specific rates of cancer incidence by age group were calculated. Age-standardized rates (ASRs) were determined using the world standard population. Changes in the annual ASRs were examined by calculating the annual percentage change (APC) over a time period as (exp(β)-1) ×100, where the regression coefficient (β) was estimated from a linear regression between logarithmic-transformed age-standardized cancer rates [24].

Long-term trends by age group and histologic type were analyzed using the joinpoint regression model [25]. Joinpoint regression was applied to detect significant changes in cancer rates. This method describes changes in data trends by connecting several different line segments on a log scale at “joinpoints”. The analysis starts with the minimum number of joinpoints and tests for a model fit with a maximum of three joinpoints. The Monte Carlo permutation method was used for tests of significance. The analysis was performed using Joinpoint software 4.0 from the Surveillance Research Program of the National Cancer Institute in the United States [26].

## Results

### Trends in the Invasive Cervical Cancer and Incidence of *in* *situ*

**Figure 1:**
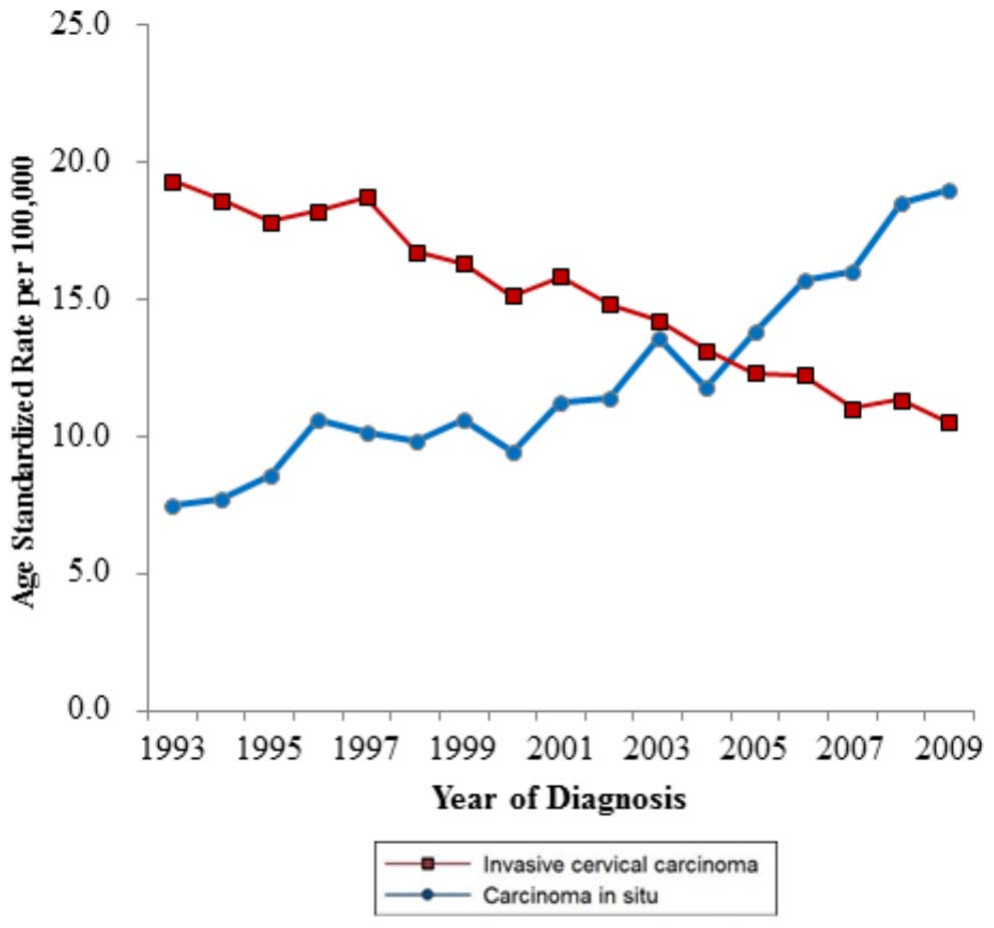
Treads in age standardized *in situ* and invasive cervical carcinoma rates and Ratio of age-standardized invasive to *in situ* cervical carcinoma rates, Korea, 1993–2009.

72,240 cases of ICC were diagnosed (Tables 1). Total incidence rate of ICC was 14.7 per 100,000 females from 1993 to 2009. ASRs for ICC decreased from 19.3 per 100,000 in 1993 to 10.5 per 100,000 in 2009 (APC: −4.2 [95% confidence interval (CI): −4.7, −3.7]) (Figure 1). The main morphologic type of ICC was squamous cell carcinoma (80.6%), but the proportion of adenocarcinoma increased from 8.9% in 1993 to 16.1% in 2009. Although, the cases and proportion of adenocarcinoma increased from 383 in 1993 to 602 in 2009, ASRs for adenocarcinoma did not increase significantly (APC: 0.6 [95% CI: −0.2, 1.4]).

**Table 1:** Numbers and age standardized rates of invasive cervical cancer by year of diagnosis, and histologic type, Korea, 1993–2009.

Year of diagnosis	Histologic type							
	Squamous cell carcinoma		Adenocarcinoma		Others & Unknown		Total	
	Cases (%)	ASR^1^	Cases (%)	ASR^1^	Cases (%)	ASR^1^	Cases (%)	ASR^1^
1993	3,455 (80.0)	15.4	383 (8.9)	1.7	481 (11.1)	2.2	4,319 (100.0)	19.3
1994	3,600 (83.4)	15.5	347 (8.0)	1.5	371 (8.6)	1.6	4,318 (100.0)	18.6
1995	3,598 (84.0)	15.0	326 (7.6)	1.4	360 (8.4)	1.5	4,284 (100.0)	17.8
1996	3,697 (81.7)	14.8	430 (9.5)	1.8	398 (8.8)	1.6	4,525 (100.0)	18.2
1997	3,978 (83.0)	15.5	471 (9.8)	1.7	345 (7.2)	1.4	4,794 (100.0)	18.7
1998	3,642 (82.3)	13.8	464 (10.5)	1.6	317 (7.2)	1.2	4,423 (100.0)	16.7
1999	3,629 (81.7)	12.2	447 (10.1)	1.6	367 (8.3)	1.3	4,443 (100.0)	16.3
2000	3,436 (80.8)	12.9	445 (10.5)	1.9	372 (8.7)	1.3	4,253 (100.0)	15.1
2001	3,728 (81.5)	12.0	545 (11.9)	1.6	299 (6.5)	1.0	4,572 (100.0)	15.8
2002	3,549 (80.6)	11.4	485 (11.0)	1.8	368 (8.4)	1.2	4,402 (100.0)	14.8
2003	3,492 (80.1)	10.2	556 (12.7)	2.0	313 (7.2)	1.0	4,361 (100.0)	14.2
2004	3,187 (77.3)	11.4	615 (14.9)	1.8	321 (7.8)	1.0	4,123 (100.0)	13.1
2005	3,109 (77.8)	10.2	566 (14.2)	1.7	320 (8.0)	0.9	3,995 (100.0)	12.4
2006	3,158 (78.4)	9.7	610 (15.1)	1.8	261 (6.5)	0.8	4,029 (100.0)	12.2
2007	2,932 (78.6)	9.6	572 (15.3)	1.7	225 (6.0)	0.6	3,729 (100.0)	11.0
2008	3,123 (79.3)	8.7	593 (15.1)	1.7	221 (5.6)	0.6	3,937 (100.0)	11.3
2009	2,889 (77.4)	9.0	602 (16.1)	1.7	242 (6.5)	0.6	3,733 (100.0)	10.5
EAPC^2^ (95% CI)	−4.2 (−4.7, −3.7)		0.6 (−0.2, 1.4)		−6.8 (−7.9, −5.7)		−3.8 (−4.3, −3.4)	
1993–2009	58,202 (80.6)	11.8	8,457 (11.7)	1.7	5,581 (11.7%)	1.1	72,240 (100.0)	14.7

1 ASR: Age-standardized rates.

2 EAPC: Estimated annual percent change.

Between 1993 and 2009, 62,300 cases of CIS were reported in Korea (Table 2). Total incidence rate of CIS was 12.2 per 100,000 females from 1993 to 2009. ASRs for CIS increased from 7.5 per 100,000 females in 1993 to 19.0 per 100,000 females in 2009 (APC: 5.7 [95% CI: 4.8, 6.7]) (Figure 1). Most CIS cases diagnosed from 1993 to 2009 were squamous cell type (96.3%). Only 1.7% of CIS cases were adenocarcinoma in situ, and 2.1% were reported as other or unspecified cell type.

**Table 2:** Numbers and age standardized rates of in situ cervix uteri by year of diagnosis, and histologic type, Korea, 1993–2009.

Year of diagnosis	Histologic type							
	Squamous cell carcinoma		Adenocarcinoma		Others & Unknown		Total	
	Cases (%)	ASR^1^	Cases (%)	ASR^1^	Cases (%)	ASR^1^	Cases (%)	ASR^1^
1993	1,676 (92.3)	6.9	17 (0.9)	0.1	123 (6.8)	0.6	1,816 (100.0)	7.5
1994	1,820 (92.0)	7.0	20 (1.0)	0.1	138 (7.0)	0.6	1,978 (100.0)	7.7
1995	2,081 (93.4)	7.9	22 (1.0)	0.1	126 (5.7)	0.5	2,229 (100.0)	8.6
1996	2,712 (94.7)	10.0	25 (0.9)	0.1	126 (4.4)	0.5	2,863 (100.0)	10.6
1997	2,654 (94.6)	9.5	22 (0.8)	0.1	129 (4.6)	0.5	2,805 (100.0)	10.1
1998	2,692 (96.4)	9.4	29 (1.0)	0.1	71 (2.5)	0.3	2,792 (100.0)	9.8
1999	3,015 (97.5)	10.3	41 (1.3)	0.2	36 (1.2)	0.1	3,092 (100.0)	10.6
2000	2,715 (97.0)	9.1	34 (1.2)	0.1	51 (1.8)	0.2	2,800 (100.0)	9.4
2001	3,308 (97.3)	10.9	44 (1.3)	0.1	48 (1.4)	0.2	3,400 (100.0)	11.2
2002	3,424 (97.1)	11.1	49 (1.4)	0.2	52 (1.5)	0.2	3,525 (100.0)	11.4
2003	4,163 (97.3)	13.2	74 (1.7)	0.2	40 (0.9)	0.1	4,277 (100.0)	13.6
2004	3,627 (96.5)	11.4	65 (1.7)	0.2	66 (1.8)	0.2	3,758 (100.0)	11.8
2005	4,270 (96.1)	13.3	96 (2.2)	0.3	75 (1.7)	0.2	4,441 (100.0)	13.8
2006	4,898 (96.9)	15.2	108 (2.1)	0.3	47 (0.9)	0.1	5,053 (100.0)	15.7
2007	5,042 (97.2)	15.6	91 (1.8)	0.3	53 (1.0)	0.1	5,186 (100.0)	16.0
2008	5,840 (96.8)	17.9	144 (2.4)	0.4	51 (0.8)	0.1	6,035 (100.0)	18.5
2009	6,053 (96.8)	18.5	150 (2.4)	0.5	47 (0.8)	0.1	6,250 (100.0)	19.0
EAPC^2^ (95% CI)	6.0 (5.1, 6.9)		13.2 (11.4, 15.1)		−11.1 (−13.8, −8.3)		5.7 (4.8, 6.7)	
1993–2009	59,990 (96.3)	11.8	1,030 (1.7)	0.2	1,279 (2.1)	0.2	62,300 (100.0)	12.2

1 ASR: Age-standardized rates.

2 EAPC: Estimated annual percent change.

### Trends in the Incidence of *in situ* and Invasive Cervical Cancer by Age Group and Histological Type

**Figure 2:**
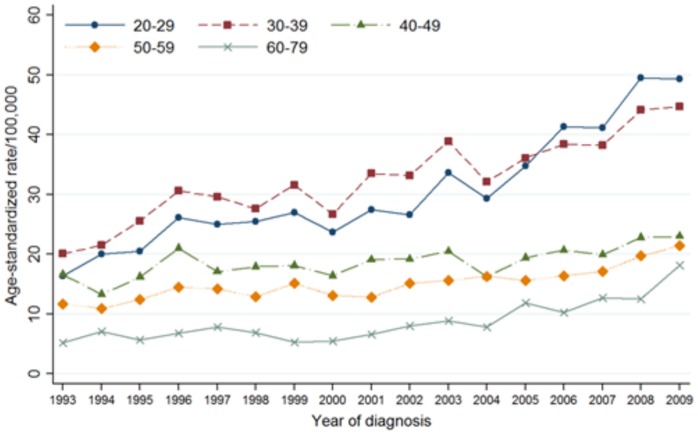
Trends in age standardized incidence rate for cervical carcinoma *in situ* according to the age group in Korea, 1993–2009.

**Figure 3:**
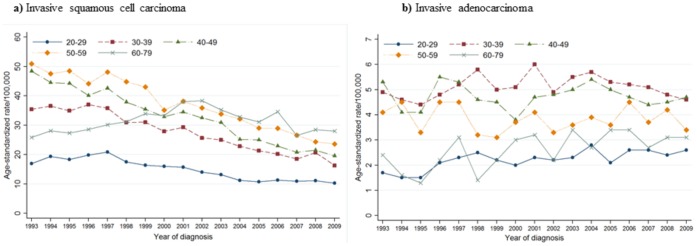
Trends in age standardized incidence rate for invasive squamous cell carcinoma and adenocarcinoma according to the age group in Korea, 1993–2009.

Table 3 and Figures 2 and 3 showed trends in the incidence of *in situ* and invasive cervical cancer by age group and histological type. For CIS, all age groups showed an increasing trend from 1993 to 2009. Except for the 60–79 age group, the incidence of CIS increased for the entire study period. For women aged 60–79 years, the incidence of CIS showed no trend from 1993 to 2000 and showed an increasing trend [APC: 11.2% (95% CI: 6.7, 15.8)] from 2000 to 2009.

**Table 3:** Joinpoint regression of incidence of *in situ* and invasive cervical cancer by histologic type and age group in Korea, 1993–2009.

Histologic type	Age group	Trend 1			Trend 2		
		Years	EAPC^1^ (95% CI)		Years	EAPC^1^ (95% CI)	
**CIS** 2							
All type	20–29	1993–2009	6.0	(4.9, 7.2)			
	30–39	1993–2009	4.0	(3.1, 5.0)			
	40–49	1993–2009	2.0	(0.9, 3.1)			
	50–59	1993–2009	3.2	(2.3, 4.1)			
	60–79	1993–2000	−0.7	(−6.5, 5.4)	2000–2009	11.2	(6.7, 15.8)
**ICC** 3							
All type	20–29	1993–2005	−0.3	(−0.7, 1.1)	2005–2009	5.7	(−1.9, 13.7)
	30–39	1993–2009	−1.7	(−2.2, −1.2)			
	40–49	1993–2009	−3.7	(−4.2, −3.3)			
	50–59	1993–2009	−3.5	(−3.9, −3.1)			
	60–79	1993–2001	4.5	(2.7, 6.3)	2001–2009	−2.6	(−4.2, −0.8)
Squamous cell carcinoma	20–29	1993–1996	4.5	(−6.6, 16.8)	1996–2009	−5.4	(−6.5, −4.3)
	30–39	1993–1996	1.0	(−6.9, 9.6)	1996–2009	−5.6	(−6.4, −4.8)
	40–49	1993–2009	−5.5	(−6.1, −5.0)			
	50–59	1993–1997	−1.5	(−5.5, 2.5)	1997–2009	−5.5	(−6.2, −4.7)
	60–79	1993–2001	4.2	(2.6, 5.8)	2001–2009	−4.6	(−6.7, −2.4)
Adenocarcinoma	20–29	1993–2001	2.8	(1.5, 4.2)			
	30–39	1993–2001	0.3	(−0.6, 1.2)			
	40–49	1993–2001	0.0	(−1.1, 1.2)			
	50–59	1993–2001	−0.4	(−1.8, 1.0)			
	60–79	1993–2001	3.9	(1.3, 6.6)			

1 EAPC: Estimated annual percent change.

2 CIS: Cervical carcinoma *in situ.*

3 ICC: Invasive cervical carcinoma.

For ICC, the incidence rate for women aged 30–59 years showed a decreasing trend from 1993 to 2009. Women aged 40–49 years showed the largest decrease [APC: −3.7% (95% confidence interval (CI): −4.2, −3.3)], followed by 50–59 years [APC: −3.5% (95% CI: −3.9, −3.1)], 30–39 years [APC: −1.7% (95% CI: −2.2, −1.2)]. The incidence rate for women aged 60–79 years increased from 1993 to 2001, and showed a decreasing trend [APC, −2.6% (95% CI: −4.2, −0.8)] from 2001 to 2009. There was no significant trend in the incidence of ICC among women aged 20–29 from 1993 to 2009.

For invasive squamous cell carcinoma, the incidence rates are generally different by different age group. While there was no significant trend in the incidence of ICC among women aged 20–29 from 1993 to 2006, there was a significant decreasing trend from 1996 to 2009 in their 20 s [APC: −5.4% (95% CI: −6.5, −4.3)]. While there was no significant trend in the incidence of ICC among women aged 30–39 from 1993 to 2006, there was a significant decreasing trend from 1996 to 2009 in their 30 s [APC: −5.6% (95% CI: −6.4, −4.8)]. While there was no significant trend in the incidence of ICC among women aged 50–59 from 1993 to 2007, there was a significant decreasing trend from 1997 to 2009 in their 50 s [APC: −5.5% (95% CI: −6.2, −4.7)]. Interestingly, for women in their 40 s, ASRs for ICC decreased during the entire study period [APC: −5.5% (95% CI: −6.1, −5.0)]. For women aged 60 or older, the ASR for squamous cell carcinoma increased 4.2% per year from 1993 to 2001, and then the ASR sharply decreased 4.6% per year from 2001 to 2009. Invasive adenocarcinoma showed an increasing trend among women aged 20–29 years [APC, 2.8% (95% CI: 1.5, 4.2)] and 60–79 years [APC, 3.9% (95% CI: 1.3, 6.6)]. On the other hands, there was no significant trend in the incidence of invasive adenocarcinoma among women aged 30–59.

### Trends in the Screening Rate for Cervical Cancer by Age Group

We additionally showed trends in the screening rate for cervical cancer of Korea by age group (Table S1). For overall age groups, screening rate for cervical cancer increased from 33.5% in 1998 to 45.5% in 2009 (APC: 2.9 [95% CI: 1.0, 4.8]). Women aged 60–79 years showed the largest increase [APC: 7.29 (95% CI: 5.3, 9.3)], followed by 20–29 years [APC, 6.6% (95% CI: 2.2, 11.1)], 50–59 years [APC, 3.9% (95% CI: 1.2, 6.7)], 40–49 years [APC, 1.6% (95% CI: 0.3, 2.9)]. However, women aged 30–39 years did not show any significant increase [APC: −0.04 (95% CI: −1.3, 1.2)].

These U shaped pattern for the APCs by age group was similar those pattern of APCs of incidence rate for CIS except for 30–39 aged women. In addition, screening rate for cervical cancer showed a strong positive correlation with incidence rate for CIS (ρ = 0.87, *p*-value = 0.03; Figure S1). Moreover, screening rate for cervical cancer showed a strong negative correlation with incidence rate for squamous ICC (ρ = −0.83, *p*-value = 0.04; Figure S2).

## Discussion

The present study confirmed that the overall incidence of ICC is declining, with an increasing incidence of CIS between 1993 and 2009 in Korea. The decreasing trend in the incidence of ICC was attributed to a decrease in the incidence of squamous cell carcinoma. These results suggested the success of cervical screening and treatment of pre-invasive squamous cell carcinoma in Korea. Indeed, there was a strong negative relationship between screening rate for squamous carcinoma and incidence rate for invasive cervical cancer. Many lines of epidemiologic evidence have proven the effectiveness of cervical cancer screening on the incidence of invasive squamous cell carcinoma in the United States [11], Canada [27], Taiwan [7], and European countries [8].

Currently, two population-based organized cancer screening programs are implemented in Korea. Papanicolaou (Pap) smear was introduced in health examinations that were serviced to employees and dependents insured by the Korea National Health Insurance (NHI) Program since 1988 [28]. The NHI Cancer Screening Program (NHICSP), which provides services to the upper 50% of NHI beneficiaries by the NHI Corporation, was expanded to include all employees and dependents in 1996, and self-employees and their dependents in 2000 [29]. In 1999, the National Cancer Screening Program (NCSP) for cervical cancer administered by the Ministry of Health and Welfare was established free of charge to target populations such as Medical Aid Program recipients and the lower 50% of NHI beneficiaries [30]. These two programs provide a screening test free of charge to all women aged 30 and over biennially. These stepwise expansions of cervical screening programs in Korea caused the different starting year of the decreasing incidence of ICC, particularly SCC among different age groups. Squamous cell carcinoma showed a decreasing trend from 1993 in women in their 40 s, in the mid-1990s (1996–1997) in women aged 20–39 years, and in women in their 50 s. For women aged 60–79 years, the ASR for squamous cell carcinoma increased from 1993 to 2001 and sharply decreased from 2001 to 2009. Expanding cervical screening programs to self-employees and their dependents in 2000 might have caused the decreasing trend among women aged 60–79 years since 2001. Most women aged 60–79 years were not employed, so they could participate in the cervical screening program since 2000.

While the overall incidence rate of adenocarcinoma remained stable, it increased in women aged 20–29 years (APC 2.8%) and in women aged 60–79 years (APC 3.9%) along with the increasing incidence rate of adenocarcinoma *in situ* (APC, 13.2%), although the number of cases was small. Some reports have indicated that the incidence of adenocarcinoma has increased for the entire population in the United States [9] and European countries [14] although cervical screening programs were implemented. Some countries such as Canada [12] and the Netherlands [13] have shown an increasing incidence of adenocarcinoma only for young women. The reason for the increasing incidence of adenocarcinoma may be due to less success in diagnosing [31]–[34] and treating pre-invasive adenocarcinoma, as compared to squamous cell carcinoma [33], [35]. The accuracy of the Pap smear for detecting cervical adenocarcinoma was lower than that for squamous cell carcinoma because adenocarcinoma was detected in the endocervical canal, which is difficult to find relative to squamous cell carcinoma [31], [32].

Recent-birth cohort groups have a greater risk than those born before 1935 in European countries, and this finding can be explained by increased HPV infection through the change in sexual behavior [13]. In Korea, the proportion of abnormal Pap smear results are highest in younger age groups, indicating that younger people have different sexual habits compared with older cohorts [36]. Additionally, the overall prevalence rate of high-risk HPV was 16.7% [37], which was over 10% higher than the average prevalence rate of high-risk HPV worldwide [38], and there was a report that the prevalence rate of HPV showed an increased trend with decreasing age [23], [39].

Our study showed that cervical adenocarcinoma increased among women aged 60–79 years as well as among women aged 20–29 years. This significant increase in women aged 60–79 years may be an artifact. Because the incidence rate of adenocarcinoma was relatively low, the APC of adenocarcinoma could show a statistically significant result only if some cases were added. Actually, cases of adenocarcinoma among women aged 60–79 years increased from 70 cases in 1993 to 117 cases in 2009. As screening participants aged 60–79 years increased, enhancing the validity of registry data, incidence of adenocarcinoma could be increased. The other possible reason for increasing adenocarcinoma was supposed to an increased HPV prevalence especially among the young women.

In addition, there was a strong positive correlation between screening rate for cervical cancer and incidence rate for CIS, whereas screening rate showed a strong negative correlation with incidence rate for squamous ICC. These results may suggest that the incidence rate of all types of cervical cancer may be affected by the screening rate, despite the low accuracy of screening for adenocarcinoma.

Our study possesses several limitations. First, although invasive cancer data met the standards of quality required for inclusion in the cancer incidence in five continents [17], this has not been evaluated for CIS. However, we collect cancer data, including CIS data, from hospitals passively and through active survey as well, suggesting that data completeness and quality may be high for both invasive and *in situ* cancers. Second, there may be differences in completeness between 1993–1998 and 1999–2009. The KCCR constructed the KNCI DB from 1999. Therefore, an improvement of completeness might lead us to overestimate the increasing trend of CIS or underestimate the decreasing trend of invasive cancer. We also conducted our analysis from 1993 because we also used data from the Gynecologic Oncology Committee of the Korean Society of Obstetrics and Gynecology, which began a gynecologic cancer registry in 1991. In addition, if there were some underreporting cases of ICC from 1993 to 1998, we can expect to observe more decrease than result of this study. Third, the incidence rate of adenocarcinoma was relatively low, and the trend in the incidence of adenocarcinoma has fluctuated in Korea. Thus, it seems too early to conclude a trend for adenocarcinoma. Over time, the accuracy of classification of histological type has been improved with decreasing proportion of others and unspecified cell type, However, it was impossible to explain that an increase in squamous cell CIS or adenocarcinoma in situ or decrease in invasive SCC were attributable to only a decreased proportion of others or unspecified cell type. Therefore, we suggested that there was a real increase in squamous cell or adenocarcinoma CIS and decrease in invasive SCC.

In conclusion, the present study showed that cervical CIS has increased, and cervical squamous cell carcinoma has decreased among Korean women since 1993. Invasive adenocarcinoma did not significantly decrease; indeed, it appears to have increased, predominantly in young Korean women.

## Supporting Information

Figure S1 **The association between screening rate for cervical cancer and incidence rate for carcinoma in situ, 1998–2009.**
(TIFF)

Figure S2 **The association between screening rate for cervical cancer and incidence rate for invasive cervical carcinoma, 1998–2009.**
(TIFF)

Table S1 **Age-standardized cervical cancer screening rates and annual percent change (APC) according to the age group in Korea, 1998–2011.**
(DOCX)
