# Supplementary figures and images for: Trends in the Incidence of In Situ and Invasive Cervical Cancer by Age Group and Histological Type in Korea from 1993 to 2009
Source: PLoS One. 2013 Aug 16;8(8):e72012. doi: 10.1371/journal.pone.0072012 (PMC3745377; doi:10.1371/journal.pone.0072012)

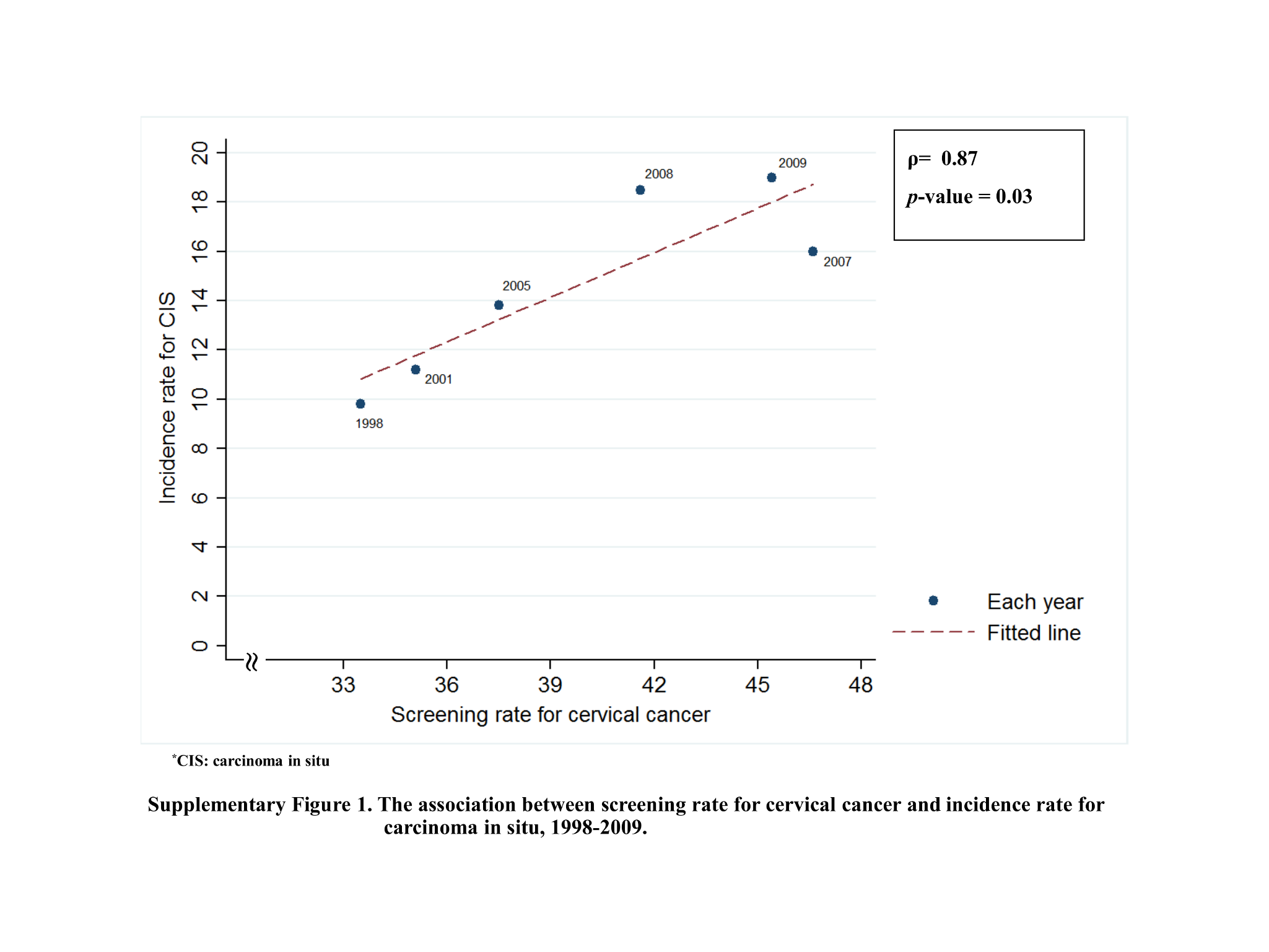

Supplement: Figure S1 — The association between screening rate for cervical cancer and incidence rate for carcinoma in situ, 1998–2009. (TIFF) [file pone.0072012.s001.tiff]

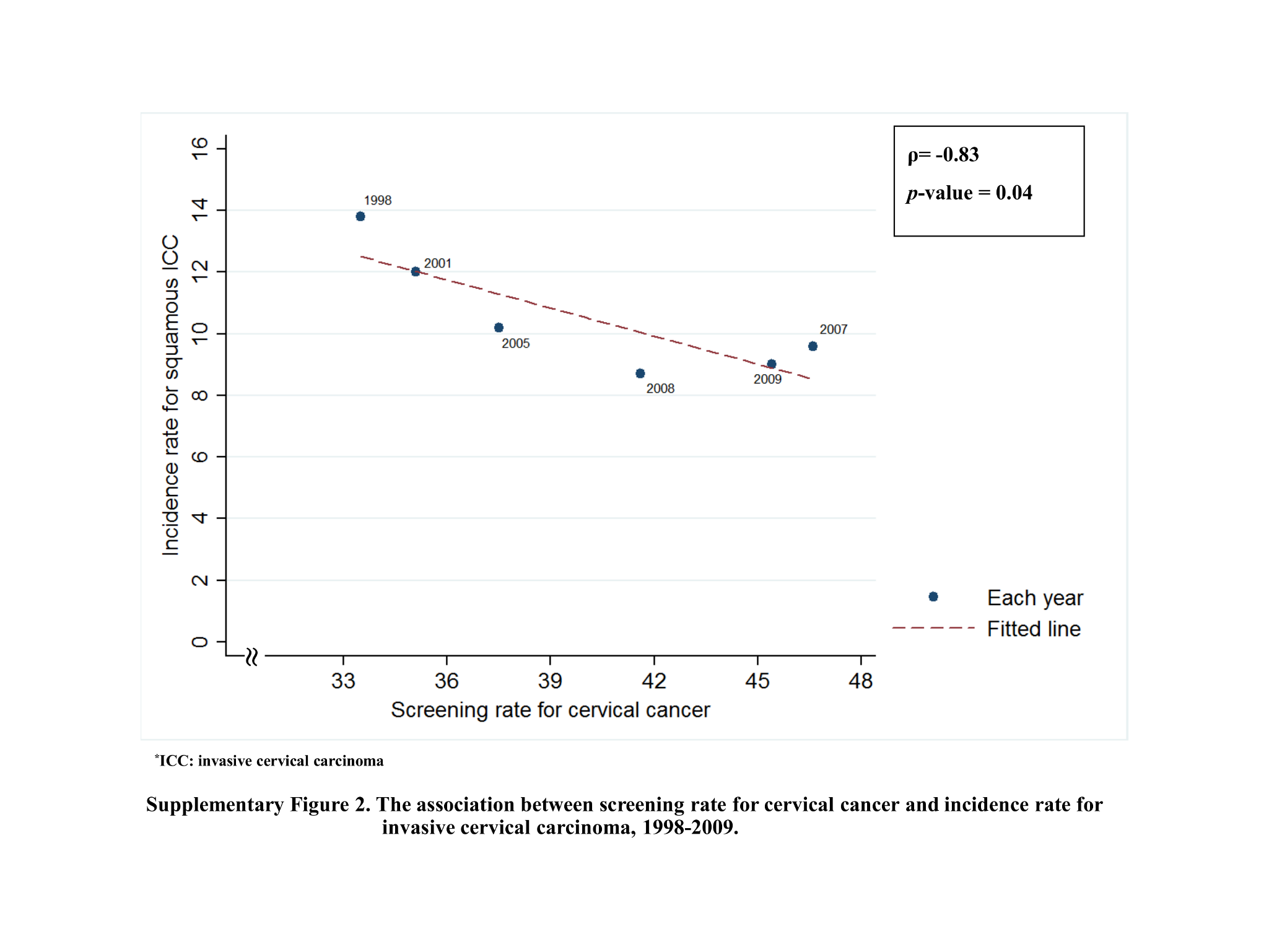

Supplement: Figure S2 — The association between screening rate for cervical cancer and incidence rate for invasive cervical carcinoma, 1998–2009. (TIFF) [file pone.0072012.s002.tiff]
