# Supplementary material for: Trends in the Incidence of In Situ and Invasive Cervical Cancer by Age Group and Histological Type in Korea from 1993 to 2009
Source: PLoS One. 2013 Aug 16;8(8):e72012. doi: 10.1371/journal.pone.0072012 (PMC3745377; doi:10.1371/journal.pone.0072012)
Supplement: Table S1 — Age-standardized cervical cancer screening rates and annual percent change (APC) according to the age group in Korea, 1998–2011. (DOCX) [file pone.0072012.s003.docx]

**Supplementary table 1. Age-standardized cervical cancer screening rates and annual percent change (APC) according to the age group in Korea, 1998–2011**

| **Age group** | **Year** | | | | | | **APC (95% CI)^*^** |
| --- | --- | --- | --- | --- | --- | --- | --- |
|  | **1998** | **2001** | **2005** | **2007** | **2008** | **2009** |  |
|  |  |  |  | | | |  |
| 20-29 | 13.8 | 15.0 | 18.9 | 30.0 | 24.3 | 24.3 | 6.6 (2.2, 11.1)^*^ |
| 30-39 | 50.4 | 48.9 | 49.3 | 52.6 | 46.9 | 50.4 | -0.04 (-1.3, 1.2) |
| 40-49 | 52.8 | 52.8 | 55.1 | 62.7 | 58.1 | 62.9 | 1.6 (0.3, 2.9)^*^ |
| 50-59 | 38.8 | 43.3 | 44.0 | 60.9 | 53.6 | 59.3 | 3.9 (1.2, 6.7)^*^ |
| 60-79 | 17.1 | 21.8 | 25.9 | 33.7 | 32.4 | 39.3 | 7.3 (5.3, 9.3)^*^ |
| Overall | 33.5 | 35.1 | 37.5 | 46.6 | 41.6 | 45.4 | 2.9 (1.0, 4.8)^*^ |

**CI: Confidence intervals**

**Data was obtained from Korea National Health and Nutritional Examination Survey 1998–2011.**

**The standard population was defined as the world standard population.**

**Screening rates for cervical cancer was defined as the proportion of participants who undergone in cervical cancer screening within 2 years.**

**^*^Annual percent changes (APC) were expressed as (exp(β)-1)x100 and 95% confidence interval.**
